# Significant Efficacy of a Single Low Dose of Primaquine Compared to Stand-Alone Artemisinin Combination Therapy in Reducing Gametocyte Carriage in Cambodian Patients with Uncomplicated Multidrug-Resistant Plasmodium falciparum Malaria

**DOI:** 10.1128/AAC.02108-19

**Published:** 2020-05-21

**Authors:** Amélie Vantaux, Saorin Kim, Eakpor Piv, Sophy Chy, Laura Berne, Nimol Khim, Dysoley Lek, Sovannaroth Siv, Mavuto Mukaka, Walter R. Taylor, Didier Ménard

**Affiliations:** a Malaria Molecular Epidemiology Unit, Institut Pasteur of Cambodia, Phnom Penh, Cambodia; b National Center for Parasitology, Entomology and Malaria Control Program, Phnom Penh, Cambodia; c School of Public Health, National Institute of Public Health, Phnom Penh, Cambodia; d Mahidol Oxford Tropical Medicine Research Unit, Bangkok, Thailand; e Centre for Tropical Medicine and Global Health, Nuffield Department of Medicine, University of Oxford, Oxford, United Kingdom

**Keywords:** direct membrane feeding assays, transmission blocking, malaria, primaquine

## Abstract

Since 2012, a single low dose of primaquine (SLDPQ; 0.25 mg/kg of body weight) with artemisinin-based combination therapies has been recommended as the first-line treatment of acute uncomplicated Plasmodium falciparum malaria to interrupt its transmission, especially in low-transmission settings of multidrug resistance, including artemisinin resistance. Policy makers in Cambodia have been reluctant to implement this recommendation due to primaquine safety concerns and a lack of data on its efficacy. In this randomized controlled trial, 109 Cambodians with acute uncomplicated P. falciparum malaria received dihydroartemisinin-piperaquine (DP) alone or combined with SLDPQ on the first treatment day. The transmission-blocking efficacy of SLDPQ was evaluated on days 0, 1, 2, 3, 7, 14, 21, and 28, and recrudescence by reverse transcriptase PCR (RT-PCR) (gametocyte prevalence) and membrane feeding assays with Anopheles minimus mosquitoes (gametocyte infectivity). Without the influence of recrudescent infections, DP-SLDPQ reduced gametocyte carriage 3-fold compared to that achieved with DP. Of 48 patients tested on day 0, only 3 patients were infectious to mosquitoes (∼6%). Posttreatment, three patients were infectious on day 14 (3.5%, 1/29) and on the 1st and 7th days of recrudescence (8.3%, 1/12 for each); this overall low infectivity precluded our ability to assess its transmission-blocking efficacy. Our study confirms the effective gametocyte clearance of SLDPQ when combined with DP in multidrug-resistant P. falciparum infections and the negative impact of recrudescent infections due to poor DP efficacy. Artesunate-mefloquine (ASMQ) has replaced DP, and ASMQ-SLDPQ has been deployed to treat all patients with symptomatic P. falciparum infections to further support the elimination of multidrug-resistant P. falciparum in Cambodia. (This study has been registered at ClinicalTrials.gov under identifier NCT02434952.)

## Abstract (precis)

Since 2012, a single low dose of primaquine (SLDPQ; 0.25 mg/kg of body weight) with artemisinin-based combination therapies has been recommended as the first-line treatment of acute uncomplicated Plasmodium falciparum malaria to interrupt its transmission, especially in low-transmission settings of multidrug resistance, including artemisinin resistance. Policy makers in Cambodia have been reluctant to implement this recommendation due to primaquine safety concerns and a lack of data on its efficacy.

## INTRODUCTION

Malaria remains a major public health challenge, with an estimated 228 million cases recorded in 2018 (1). While considerable progress has been made since 2010, when the estimated case burden was 251 million, the recent emergence and spread of the Plasmodium falciparum lineage resistant to both artemisinin and piperaquine (the KEL1/PLA1 lineage) in the eastern Greater Mekong Subregion (GMS) threaten this remarkable global achievement (2–7). Eliminating rapidly multidrug-resistant (MDR) Plasmodium falciparum is therefore the top priority for countries of the GMS. Implementation of novel tools and strategies or repurposing existing tools, like primaquine, that specifically aim at interrupting malaria transmission are essential to reach this goal.

Although artemisinin derivatives are the more potent compounds in artemisinin-based combination therapies (ACTs) and are active against early gametocyte stages (8–10) and reduce transmission (11, 12), the only currently available drug effective against the transmissible mature stage V gametocytes is primaquine. In 2012, the WHO recommended the addition of a single low dose of primaquine (SLDPQ; target dose, 0.25 mg/kg of body weight) to ACTs to interrupt malaria transmission, primarily in areas with low rates of transmission of MDR P. falciparum, like GMS (13).

To date, several clinical studies conducted in Africa, Colombia, and Cambodia have assessed the safety and efficacy of SLDPQ (14–24), mainly by measuring gametocyte carriage (by microscopy or TaqMan reverse transcriptase PCR [RT-PCR]) as a surrogate marker of its transmission-blocking efficacy. This is due to the challenging logistical requirements of carrying out mosquito infectivity studies. These studies showed that SLDPQ, dosed from 0.20 to 0.75 mg/kg, clearly reduced gametocyte carriage and increased gametocyte clearance in a dose-dependent manner (15–17, 19, 21–25). A small number of studies have investigated human-to-mosquito transmission, based on infectivity measures, with variable results being found due to low infectivity before and/or after SLDPQ treatment (16, 17, 19, 22, 24). Only two studies from Mali demonstrated conclusively the transmission-blocking efficacy of SLDPQ, which produced a 92.6% to 100% within-person reduction in infectivity at day 2 versus that at baseline following treatment with 0.25 mg/kg of SLDPQ (16, 19).

In Cambodia, glucose-6-phosphate dehydrogenase deficiency (G6PDd) is a common X-linked disorder of the red blood cells, with frequencies ranging from 10.8% to 29.6% in males (26) and with a prevalence rate in malaria patients seen at health centers of 13.9% (27). This is the main reason why Cambodian policy makers have been reluctant to deploy SLDPQ without local data on the safety and the efficacy of SLDPQ. Therefore, we conducted a trial assessing the tolerability and the safety of SLDPQ (0.25 mg/kg) in Cambodia in 2015 and 2016 (28), which followed on from a safety trial of 0.75 mg/kg/week of primaquine (PQ) in vivax malaria patients (29). Here, we present the results of our investigations on the transmission-blocking efficacy of this treatment through the evaluation of gametocyte prevalence dynamics as well as infectivity measures for a subset of patients using membrane feeding assays with Anopheles minimus mosquitoes, one of the main malaria vectors in Southeast Asia.

## RESULTS

One hundred nine patients with uncomplicated falciparum malaria were enrolled and treated with the standard 3-day regimen of dihydroartemisinin-piperaquine (DP) alone (48.6%, 53/109) or DP-SLDPQ (51.4%, 56/109). Seven patients were lost to follow-up (6.4%). Of the remaining 102 patients, 28 (27.4%) had PCR-proven P. falciparum recrudescences; 24 occurred within the 28-day follow-up on days 12 (*n = *1), 14 (*n = *1), 17 (*n = *2), 18 (*n = *1), 21 (*n = *2), 22 (*n = *2), 23 (*n = *1), 24 (*n = *2), 25 (*n = *1), 26 (*n = *2), 27 (*n = *1), and 28 (*n = *8), and 4 occurred on days 44, 46, 52, and 124. Of the 28 patients with recrudescences, 10 were retreated with the standard 3-day DP regimen and 18 were treated with quinine or quinine plus tetracycline; 6 of the 10 patients retreated with DP experienced a second recrudescence.

At day 0, gametocytes were detected in 47/109 patients (43.1%) by TaqMan RT-PCR (Table 1) (see reference 30 for further details), with no significant difference between the two arms (*P = *0.3057).

**TABLE 1:** Baseline characteristics of the two study groups of patients

Characteristic	Value for the following study group:	
	DP	DP-SLDPQ
No. of patients enrolled	53	56
No. of recrudescent patients	18	10
No. of males, no. of females	44, 9	44, 12
Mean (SD) age (yr)	25 (16)	26 (14)
No. of patients G6PD^*a*^ deficient, no. of patients G6PD normal	3, 50	6, 50
No. of gametocyte-positive slides	14	7
Mean (SD, range) no. of gametocytes/μl	64 (250, 0–1,432)	40 (149, 0–787)
No. of patients positive for gametocytes by TaqMan RT-PCR	26	21
Hemoglobin concn (g/dl)	12.63	13.19
No. of patients with anemia^*b*^/total no. tested	10/51	9/56

a G6PD, glucose-6-phosphate dehydrogenase.

b Anemia was considered a hemoglobin concentration of <11 g/dl.

### Gametocyte dynamics.

Examining the dynamics of TaqMan RT-PCR gametocyte carriage to day 28, drug arm, baseline gametocytemia, recrudescence status, the baseline hemoglobin concentration, and day of follow-up were all significant factors for gametocytemia in the univariate analysis (Table 2).

**TABLE 2:** Independent factors associated with a change in gametocytemia over 28 days by univariate analysis^*a*^

Factor	OR	95% CI	*P* value
Treatment			
DP-SLDPQ			
DP	5.59	1.71, 18.27	0.0044
Status on day 0			
Negative			
Positive	15.83	4.83, 51.91	<0.0001
Patient recrudescence status			
Cured			
Recrudescent	11.11	2.78, 44.36	0.0006
Day 0 hemoglobin concn	0.46	0.34, 0.63	<0.0001
Day of follow-up	0.94	0.91, 0.96	<0.0001

a OR, odds ratio (an odds ratio of <1 indicates a negative association); CI, confidence interval.

**FIG 1:**
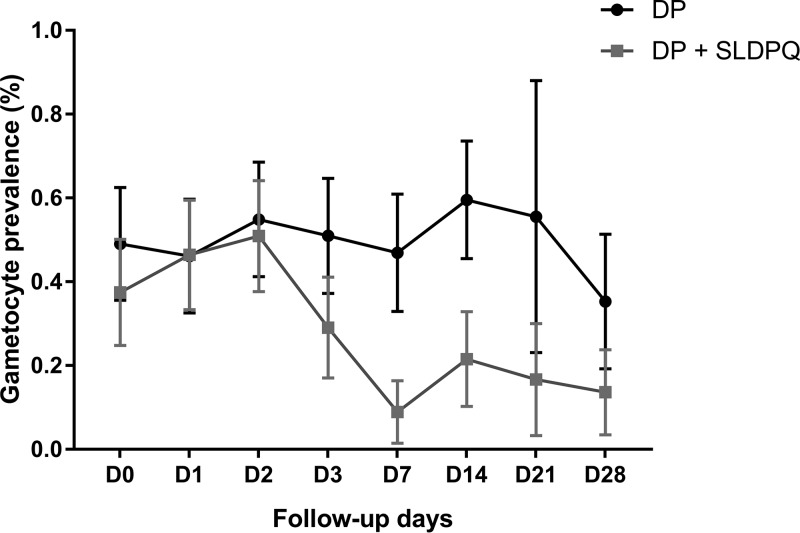
Gametocyte prevalence by TaqMan RT-PCR by follow-up day and treatment group. Data show the proportion ± 95% confidence intervals. DP, dihydroartemisinin-piperaquine.

**FIG 2:**
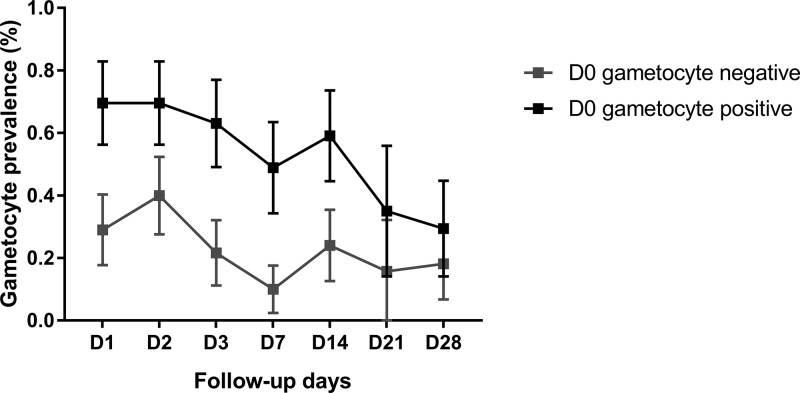
Gametocyte prevalence by TaqMan RT-PCR by follow-up day and presence of gametocyte prior to treatment on day 0 (D0), detected by TaqMan RT-PCR. Data show the proportion ± 95% confidence intervals.

The DP recipients were more likely to be gametocytemic than the DP-SLDPQ recipients during the follow-up, which was seen more clearly on day 7 and day 14 (Table 2; Fig. 1), as were patients with baseline gametocytemia (Table 2; Fig. 2) and recrudescent versus cured patients (Table 2).

The baseline hemoglobin concentration and the day of follow-up were inversely associated with the probability of being gametocytemic during the follow-up (Table 2).

When the aforementioned factors were tested in a multivariable model, all except drug arm remained significant (Table 3, model 1). However, when removing recrudescence from the multivariable model, drug arm was again a significant explanatory factor (Table 3, model 2).

**TABLE 3:** Multivariable models (with and without recrudescence) of the factors associated with changes in gametocytemia over 28 days^*a*^

Model	Factor	Adj OR	95% CI	*P* value
Model 1	Treatment	2.39	0.81, 7.08	0.1152
	Day 0 status	7.35	2.27, 23.78	0.0009
	Recrudescence	6.02	1.70, 21.26	0.0053
	Day 0 hemoglobin concn	0.57	0.42, 0.77	0.0003
	Day of follow-up	0.93	0.90, 0.96	<0.0001
Model 2	Treatment	3.15	1.09, 9.11	0.0344
	Day 0 status	6.63	2.10, 20.87	0.0012
	Day 0 hemoglobin concn	0.55	0.41, 0.75	0.0001
	Day of follow-up	0.93	0.90, 0.96	<0.0001

a Adj OR, adjusted odds ratio; CI, confidence interval.

*Post hoc* calculation showed that there was a 99.1% power to detect a difference between the DP and DP-SLDPQ treatments with the study sample sizes (*n* = 49 and *n* = 56 on day 7 for the DP and DP-SLDPQ treatment groups, respectively).

### Mosquito infectivity.

A total of 55 patients were included in the direct membrane feeding assays (DMFAs). Among the 292 feeding assays, only 113 of 14,444 (0.78%) dissected mosquitoes were infected (Table 4). Overall, 3 out of 48 (6.3% ± 6.8%) individuals infected at least one mosquito at baseline, one individual (treated with DP) was infectious on day 14, and two individuals with recrudescent infections (Tables 4 and 5) who were repeat DP treatment failures were infectious (i) at presentation on day 124 (DP-SLDPQ) and (ii) day 35 (7 days after DP was started on day 28). The three individuals infectious on day 0 were all gametocytemic by microscopy, received DP-SLDPQ, and were not infectious on day 1 or day 3 posttreatment (Table 5).

**TABLE 4:** Infectivity rates defined by treatment assessed by DMFAs during the 28-day follow-up^*a*^

Follow-up day or recrudescence, day	No. of positive isolates^*b*^/no. of isolates tested for the following treatment:			Infectivity rate (%)
	DP	DP-SLDPQ	Total	
0	0/15	3/33	3/48	6.3 ± 6.8
1	0/1	0/6	0/7	0
2	0/12	0/26	0/38	0
3	0/15	0/31	0/46	0
7	0/11	0/24	0/35	0
14	1/10	0/19	1/29	3.5 ± 6.6
21	0/1	0/4	0/5	0
28	0/6	0/22	0/28	0
Rec1, 0	0/4	1/8	1/12	8.3 ± 15.6
Rec1, 3	0/4	0/6	0/11	0
Rec1, 7	1/6	0/6	1/12	8.3 ± 15.6
Rec1, 14	0/4	0/1	0/4	0
Rec1, 28	NA	0/1	0/1	0
Rec1, 35	NA	0/1	0/1	0
Rec2, 0	0/4	0/1	0/5	0
Rec2, 3	0/4	0/1	0/5	0
Rec2, 7	0/4	0/1	0/5	0
				
Total DMFA	101	191	292	2 ± 1.6

a DP, dihydroartemisinin piperaquine; SLDPQ, a single low dose of primaquine; Rec1 and Rec2, the first and second recrudescences, respectively; NA, data not available.

b Positive isolates were isolates which were able to infect at least one mosquito from a direct membrane feeding assay (DFMA).

**TABLE 5:** Details of the five patients infectious for A. minimus by DMFAs during the 28-day follow-up^*a*^

Day or recrudescence, day	Patient identifier	Treatment	Sex	Age (yr)	Gametocytemia (no. of gametocytes/μl)	Gametocyte detection by TaqMan RT-PCR	Infection prevalence (proportion of infected females ± 95% CI)	Infection intensity (mean no. of oocysts ± SE)
0	D_002	DP-SLDPQ	F	29	562	Positive	41.7 ± 14	4.7 ± 0.97
1					336	Positive	0	NA
2					48	Positive	0	NA
3					0	Positive	0	NA
7					NA	Negative	0	NA
14					NA	Negative	0	NA
21					NA	Negative	0	NA
28					0	Positive	0	NA
Rec1, 0 (124)^*b*^	D_002	QN-TE			24	Positive	10 ± 8.3	2.6 ± 1.4
Rec1, 3 (127)					64	Positive	0	NA
Rec1, 7 (131)					168	Positive	0	NA
0	N_015	DP-SLDPQ	M	23	787	Positive	16 ± 10	2.12 ± 0.4
1					2,208	Positive	0	NA
2					40	Positive	0	NA
3					176	Positive	0	NA
7					NA	Negative	0	NA
14					NA	Negative	0	NA
28					NA	Negative	0	NA
Rec1, 0 (28)	N_035	QN	F	8	1,823	Positive	0	NA
Rec1, 3 (31)					11,912	Positive	0	NA
Rec1, 7 (35)					7,904	Positive	94.3 ± 7.7	82.6 ± 5.5
0	N_046	DP	M	8	1,432	Positive	0	NA
2					2,904	Positive	0	NA
3					3,952	Positive	0	NA
7					1,952	Positive	0	NA
14					432	Positive	6 ± 6.5	1
28					16	Positive	0	NA
0	N_074	DP-SLDPQ	M	25	540	Positive	86.3 ± 9.4	11.3 ± 1.7
3					8	Positive	0	NA

a QN-TE, quinine plus tetracycline; Rec1, the first recrudescence; CI, confidence interval; NA, data not available; F, female; M, male.

b The day after initial infection is indicated in parentheses.

## DISCUSSION

In 2012, the WHO recommended adding 0.25-mg/kg SLDPQ to first-line treatments (ACTs) to kill mature gametocytes and block human-to-mosquito transmission (13). As no data were available at that time in Cambodia, we investigated the transmission-blocking efficacy of SLDPQ when given with DP (the recommended first-line standard ACT in 2015) by evaluating gametocyte prevalence dynamics (by TaqMan RT-PCR) and Anopheles minimus infectivity by DMFA.

Our results showed that SLDPQ, when given on the first day of the 3-day standard ACT treatment, significantly decreased gametocyte carriage over time, starting on day 3, and that gametocyte carriage was the lowest on day 7, resulting in a 5.59-fold reduced risk relative to that with treatment with DP alone over 28 days in the univariate analysis. This finding is consistent with recent reports on the efficacy of PQ in reducing gametocyte carriage (15–17, 19, 21–25, 31) and earlier reports showing a lag between reduced gametocyte carriage and rapid mosquito infectivity. The multivariable analysis revealed the significant effect of recrudescent (resistant) infections on gametocyte carriage, with a 3-fold reduction in gametocyte carriage in the absence of resistance being seen.

Our patients had a high rate of baseline gametocytemia (19% by microscopy, just under 45% by TaqMan RT-PCR), which is similar to that reported previously in 2010 in patients with artemisinin-resistant P. falciparum infections in western Cambodia (∼19%) but which is 3-fold higher than the 6% reported earlier in Ratanakiri Province, Cambodia (32). Given the high prevalence of *PfKelch13* mutant P. falciparum parasites (63%) in our study and the very high recrudescent rate (~28%), our high gametocyte carriage rate is consistent with the presence of artemisinin-resistant P. falciparum, which has also led to piperaquine resistance (7). This is consistent with another study in Cambodia which showed that DP-treated patients from Oddar Meanchey province (northern Cambodia) also had prolonged gametocyte carriage that was seen in patients with recrudescent infections but was independent of the slow asexual parasite clearance time due to artemisinin-resistant P. falciparum (22, 35).

However, prolonged gametocyte carriage has also been seen in DP-treated African patients without artemisinin-resistant P. falciparum infection who, nevertheless, had low rates of human-to-mosquito transmission (16, 32, 33). Moreover, several comparative studies have found that gametocyte carriage is higher in DP-treated patients than in artemether-lumefantrine-treated patients (33–36), suggesting a reduced gametocytocidal activity of piperaquine compared to lumefantrine.

Overall, we observed a biphasic pattern of gametocyte prevalence, with a drop on day 7 and a rebound on day 14, followed by a decrease on day 21 and day 28. However, this effect was mainly driven by the DP-treated patients, as their gametocyte carriage rate was similar over the 28-day follow-up, whereas it decreased markedly in the DP-SLDPQ-treated patients (Fig. 1).

We were not able to assess the transmission-blocking efficacy of SLDPQ due to the very low infectivity rate observed in only three patients (∼6%) at baseline and three infectious events during follow-up. This is a major limitation of DFMAs, as previously reported by Lin et al. (22) at baseline, using Anopheles dirus mosquitoes, and on day 7, without assessing the baseline, by Okebe et al. (24). In another study in African children, despite a higher infectivity rate at enrollment after microscopy-based selection of gametocyte-positive asymptomatic children (38%), Gonçalves et al. (17) were also unable to detect the added value of SLDPQ due to very low infectivity posttreatments in all arms. As our clinical trial aimed primarily at assessing SLDPQ tolerability and safety, we did not particularly select patent or high-density gametocyte carriers and thus would have needed substantially more individuals to assess transmission-blocking efficacy. Only two studies, which were conducted in Mali, have statistically confirmed the high anti-infectivity efficacy (>90%) at 48 h of SLDPQ, when dosed at baseline, after selecting gametocyte-positive participants by microscopy (16, 19). However, we observed that the three SLDPQ-treated infectious patients on day 0 did not infect mosquitoes on subsequent days, despite the presence of gametocytes. Several reasons may explain this finding. Following primaquine treatment, the anti-infectivity effect is rapid (≤24 h) and precedes the decline in gametocytemia (37). Male gametocytes seem to be more sensitive to primaquine than female gametocytes (38), and the P. falciparum sex ratio is biased toward females, potentially resulting in delayed gametocyte clearance. The relationship between gametocytemia and infectivity is nonlinear and is, thus, an indirect measure of infectivity (39–41).

To conclude, our study shows that the addition of SLDPQ to ACT treatment for symptomatic falciparum malaria decreases substantially gametocyte carriage in patients with MDR P. falciparum infections. Although we were unable to reconfirm the findings of the elegant studies of Dicko et al. (16, 19), SLDPQ, dosed on the first day of treatment, is likely to further decrease the transmission potential of P. falciparum patients in our setting when combined with an effective ACT.

Owing to the high failure rate of DP, Cambodia has switched to artesunate-mefloquine treatment as the first-line treatment for uncomplicated P. falciparum and in January 2018 deployed SLDPQ in the whole country, partly on the basis of the findings of the trial described here and the good tolerability of SLDPQ in G6PDd patients (28). An age-based dosing regimen of SLDPQ, designed for Cambodia, will aid elimination efforts in areas where weighing scales are unavailable (42).

## MATERIALS AND METHODS

### Study design.

This open-label randomized controlled trial assessing the tolerability and the safety of SLDPQ was carried out in Banlung, Rattanakiri Province, in northeastern Cambodia in 2015 and 2016 (ClinicalTrials.gov registration number NCT02434952), as described by Dysoley et al. (28). Briefly, 109 nonpregnant, non-breast-feeding patients aged ≥1 year with acute uncomplicated falciparum malaria (≥1 asexual parasites/500 white blood cells, equivalent to 16 asexual parasites/μl) and a hemoglobin concentration of ≥6 g/dl were recruited to receive either dihydroartemisinin-piperaquine (DP; dihydroartemisinin at 40 mg and piperaquine at 320 mg; Duo-Cotecxin; Zhejiang Holley Nanhu Pharmaceutical Co. Ltd., Jiaxing, Zhejiang, China) alone or combined with SLDPQ (0.25 mg/kg given with the first dose of DP, 15 mg of primaquine base; Thai Government Pharmaceutical Organization). Clinical and laboratory assessments were performed on days 0, 1, 2, 3, 7, 14, 21, and 28. Anopheles minimus mosquitoes were fed on blood samples collected from a subset of 55 patients and on the days of recurrent (Drec) falciparum parasitemia (defined by the WHO as late treatment failures), depending on mosquito availability, using direct membrane feeding assays (DMFAs).

The sample size calculation was based on the primary outcome of a reduction in the mean day 7 hemoglobin (Hb) concentration of 1 g/dl in the DP-SLDPQ arm with G6PDd patients versus that in the DP-SLDPQ arm with patients with normal G6PD levels. Assuming a mean ± standard deviation Hb concentration of 11.27 ± 1.74 g/dl (calculated from a Southeast Asian database of ~6,800 ACT-treated, P. falciparum-infected patients of all ages [W. R. Taylor, unpublished data]), a two-sided alpha value of 0.05, and a power of 80%, the sample size was 48 patients per arm, and this number was rounded up to 50. G6PD status was initially diagnosed in the field using a fluorescent spot test to allow for the allocation of SLDPQ. The results of the qualitative Carestart (AccessBio, Somerset, NJ) rapid diagnostic test were assessed in parallel.

### Mosquito infection.

DMFAs were carried out to assess individual malaria infectivity as described previously (30). Briefly, 3- to 5-day-old A. minimus female mosquitoes were fed through membranes on the patients’ blood. The mosquitoes were starved for 24 h before being provided a blood meal. Venous blood samples were collected in heparinized tubes, and 400 μl of blood was made available in membrane feeders maintained at 37°C. Females were fed only once on freshly drawn blood. Postfeeding, unfed females were discarded and fed females were kept in cages (20 by 20 by 20 cm) with constant access to a 10% sucrose solution. Patient infectivity was determined by assessing infection prevalence (i.e., the proportion of infected females) and infection intensity (i.e., the number of *Plasmodium* oocysts in infected females). Midguts were dissected in a 1% mercurochrome stain, and the presence and the number of oocysts were determined under a microscope (×20 magnification). Dissections were performed on 7 day after the blood meal. The mean number of dissected females was 49 (range, 15 to 85; median, 50).

### Biological investigations.

Parasite RNA was extracted from TRIzol reagent (Life Technologies Holdings Pte. Ltd., Singapore)-conserved whole-blood samples using a QIAamp RNA blood minikit (Qiagen, Germany), following the protocol recommended by the manufacturer. A two-step semiquantitative real-time PCR was performed to detect malaria parasites, as previously described (43). Following PCR amplification, P. falciparum-positive samples were analyzed for the presence of gametocytes by a TaqMan RT-PCR, using primers spanning an exon-exon junction and targeting the Plasmodium falciparum meiotic recombination protein DMC1-like protein gene (GenBank accession number AF356553), as described previously (44). Gametocyte dilution series based on an *in vitro*-cultured local strain were used to estimate gametocyte blood concentrations.

### Ethical statement.

Ethical approvals were obtained from the Cambodian National Ethics Committee for Health Research (approval number 0370NECHR), and the trial is registered at ClinicalTrials.gov (ClinicalTrials.gov registration number NCT02434952). The protocols conformed to the Helsinki Declaration on ethical principles for medical research involving human subjects (version 2002), and informed written consent was obtained from all volunteers.

### Statistical analyses.

Initially, univariate generalized linear mixed models (GLMMs) with a binomial distribution were fitted to model the PCR-measured gametocyte prevalence rates. In these models, follow-up days (days 1, 2, 3, 7, 14, 21, and 28), treatment (DP versus DP-SLDPQ), the presence of gametocytes prior to treatment on day 0, recrudescence status (recrudescent versus cured patient), and the hemoglobin concentration prior to treatment on day 0 were included in the model as fixed factors. The patient identifier was coded as a random factor to account for repeated measures on the same individual. The univariate analyses were followed by analyses with a multivariable GLMM to model the dynamics of gametocyte prevalence posttreatment by including all the significant factors from the univariate analysis.

Gametocyte clearance times were not compared in the study for three principal reasons: (i) of the 21 patients with microscopically detected gametocytes on day 0, only 7 had complete follow-up microscopy data, (ii) TaqMan RT-PCR-measured gametocyte densities were highly variable during the 28 days of follow-up, and (iii) by day 28, 10/34 patients with gametocytes on day 0 by TaqMan RT-PCR were still gametocytemic (*n* = 1, DP-SLDPQ arm). *P* values of <0.05 were deemed significant. All analyses were performed in R (v3.5.1) software (45).
